# Can Anonymous Posters on Medical Forums be Reidentified?

**DOI:** 10.2196/jmir.2514

**Published:** 2013-10-03

**Authors:** Victoria Bobicev, Marina Sokolova, Khaled El Emam, Yasser Jafer, Brian Dewar, Elizabeth Jonker, Stan Matwin

**Affiliations:** ^1^Technical University of MoldovaChisinauMoldova, Republic Of; ^2^CHEO Research Institute, IncOttawa, ONCanada; ^3^University of OttawaOttawa, ONCanada; ^4^Privacy Analytics, IncOttawa, ONCanada

**Keywords:** privacy, personal health information, medical forums, text data mining

## Abstract

**Background:**

Participants in medical forums often reveal personal health information about themselves in their online postings. To feel comfortable revealing sensitive personal health information, some participants may hide their identity by posting anonymously. They can do this by using fake identities, nicknames, or pseudonyms that cannot readily be traced back to them. However, individual writing styles have unique features and it may be possible to determine the true identity of an anonymous user through author attribution analysis. Although there has been previous work on the authorship attribution problem, there has been a dearth of research on automated authorship attribution on medical forums. The focus of the paper is to demonstrate that character-based author attribution works better than word-based methods in medical forums.

**Objective:**

The goal was to build a system that accurately attributes authorship of messages posted on medical forums. The Authorship Attributor system uses text analysis techniques to crawl medical forums and automatically correlate messages written by the same authors. Authorship Attributor processes unstructured texts regardless of the document type, context, and content.

**Methods:**

The messages were labeled by nicknames of the forum participants. We evaluated the system’s performance through its accuracy on 6000 messages gathered from 2 medical forums on an in vitro fertilization (IVF) support website.

**Results:**

Given 2 lists of candidate authors (30 and 50 candidates, respectively), we obtained an *F* score accuracy in detecting authors of 75% to 80% on messages containing 100 to 150 words on average, and 97.9% on longer messages containing at least 300 words.

**Conclusions:**

Authorship can be successfully detected in short free-form messages posted on medical forums. This raises a concern about the meaningfulness of anonymous posting on such medical forums. Authorship attribution tools can be used to warn consumers wishing to post anonymously about the likelihood of their identity being determined.

## Introduction

Consumers have many opportunities to share their or their family’s personal health stories online, for example, through social networks or disease-specific forums. Such sharing might include disclosing personally identifiable information (eg, names, addresses, dates) coupled with health information (eg, symptoms, treatments, medical care) [1-3]. In fact, 19% to 28% of all Internet users participate in medical online forums, health-focused groups, and communities, and visit health-dedicated Web sites [4,5]. This shared health information can potentially be seen by a larger audience because 58% of Internet users report searching for health information [6].

To protect their identity when posting sensitive information online, consumers may post anonymously. Anonymity can be achieved by using a fake identity or by using a pseudonym or nickname. However, such methods for ensuring anonymity may not be very effective. There is evidence that online consumers reuse their usernames or handles across multiple sites, which makes it easier to figure out their true identity [7]. Even if a consumer creates a unique identity for posting information on a particular medical forum, text analysis techniques can combine textual data from different forums and correlate the ones that have been written by the same author. If any of those texts has the poster’s true identity, then even the anonymous posts can be reidentified. A real-world example of such cross-site information aggregation can be found in Li et al [8]. An attacker associated 5 profiles harvested from various forums and then aggregated the posted information. The identified personal information included laboratory test results, the patient’s full name, date of birth, spouse’s name, home address, home phone number, cell phone number, 2 email addresses, and occupation.

With the emergence of user-generated Web content, authorship analysis is being increasingly applied to online messages [9,10]. The general task of authorship analysis can mean one of several types of analyses: (1) author attribution in which the system is tasked to assign an unknown text to an author from several authors’ writing examples [11], (2) author verification in which the system is tasked to determine if some text was or was not written by an author given an example of the writing of a single author [12], or (3) author profiling in which the system is expected to identify an author’s gender, age, personality, cultural background, etc by analyzing given text written by this author [13]. Our focus in this paper is the author attribution.

These studies are characterized by a large number of candidate authors, a small volume of training and test texts, and short messages [14-19]. In Koppel et al [20], 10,000 blogs were used in the task of author detection in which 500-word snippets, one for each author, were considered test examples. Of the texts, 20% to 34% texts were classified with an average accuracy of 80%; the rest of the texts were considered unknown. In a separate study on the same dataset, a 500-word snippet was attributed to 1 of 1000 authors with coverage of 42.2% and precision of 93.2% [21]. The remaining 57.8% of snippets were considered unknown.

None of this previous work, however, dealt with messages posted on medical forums or other online venues that are dedicated to discussions of personal health information. The type of text is important because authorship attribution relies on unique characteristics of an individual’s writing style, and it cannot be assumed that one will write the same way when reviewing a fiction novel online as when asking a question about medical treatment or diagnosis.

We chose in vitro fertilization (IVF) forums that host discussions about infertility and attempts to conceive. Such discussions are very personal and it is reasonable to assume that individuals would want to participate anonymously. The website IVF.ca is an infertility outreach resource community created by patients for prospective, existing, and past IVF patients. A number of forums are maintained on the site for messages exchanging emotional support and information [22]. We did not require research ethics review for this study because all the data collected and used was from publically available sources. Our institutional research ethics board confirmed that no review of research on public datasets was necessary.

The most frequent uses of an Internet forum for infertility were sharing personal experience, provision of information or advice, expressions of gratitude/friendship, chat, requests for information, and expressions of universality (“we’re all in this together”) [23]. We applied Authorship Attributor, a new system to identify messages written by the same author, on the message contents. We used only texts posted by the authors on the forums; no metadata were used in training and testing files.

The choice of text features to analyze is one of the most influential factors in the performance of authorship attribution. The most common features used in the literature are word length [24], sentence length [25], type-token ratio, vocabulary richness [26], word and word n-grams (ie, sequences of n words) frequencies [27], and errors and idiosyncrasies [28]. These features could be obtained by using text analysis tools, such as a tokenizer (breaks a sequence of text into words, phases, etc, called tokens), sentence splitter (breaks text into sentences), lemmatizer (determines the base form for inflected words) or stemmer (reduces inflected words to their base form), and orthographic and synonym dictionaries. Syntactic features, such as parts of speech and part of speech sequences [29], chunks of text [30], syntactic dependencies of words [31], and syntactic structures [32] have been used to a lesser extent, but are still frequently applied. A part of speech tagger (assigns part of speech to each word), chunker (breaks text up into sequences of semantically related words), and syntactic parser (analyzes strings of text into their grammatical elements) are the necessary tools for obtaining these features. Some previous work used semantic features, such as synonyms and semantic dependencies [33]. These features can be obtained through specialized dictionaries and semantic parsers. In some experiments, several application-, content-, or language-specific features were applied as well. In most cases, these features were combined to obtain better results.

In this paper, we describe and evaluate a new system, Authorship Attributor, which has been constructed to crawl through medical forums and identify messages written by the same author.

## Methods

### Authorship Attribution Task

The task of authorship attribution is to identify who is the author of a text given a list of candidate authors and texts written by these candidates. Its methodology is based on a comparison of a new text to texts knowingly written by the candidates. Koppel [15] compared the accuracy of authorship attribution for a variety of feature sets and learning algorithms for a literature corpus, email, and blog posts corpora. The best accuracy (80%-86%) was obtained by support vector machine (SVM) and Bayesian regression algorithms on the basis of the 1000 most frequent words and the 1000 character trigrams with the highest information gain.

One of the most exhaustive feature sets was used by Abbasi and Chen [34]. It included characters, character bigrams and trigrams, punctuation and special characters, word length, function words, word bigrams and trigrams, vocabulary richness, part of speech tags, part of speech tag bigrams and trigrams, message length and structure, misspelled words, and other features. Experiments with this set of features showed good results: 88% to 96% accuracy (ie, correctly classified texts/all texts) for various datasets including eBay comments, a Java forum, and email and chat corpora.

Narayanan et al [10] reused this feature set but slightly changed it. Frequencies of syntactic category pairs (A, B), where A is the parent of B in the parse tree, were added to the previously described feature set. The overall number of features was approximately 1200. The authors used these features in the experiments with 100,000 blogs with an average length of 7500 words in each blog. As in all such cases, there was a trade-off between precision and recall. With a corpus of texts from 100,000 authors, the classifiers could correctly identify an anonymous author in more than 20% of cases and the correct author was one of the top 20 guesses in approximately 35% of cases. The increase in precision from 20% to more than 80% could be achieved by reducing recall in half.

In Narayanan et al [10], content-specific features (eg, keywords) positively influenced the accuracy of classification if authors were writing texts about different topics. However, many applications seek to identify authors regardless of topic [18]. Other studies have presented good results for gender and age classification [13,15]. The gender- or age-specific differences in writing can help in classification, but hide individual author-specific features. Koppel et al [21] performed a small-scale experiment using 2 authors who had posted on different topics of a listserv collection, but it was pointed out that it is extremely difficult to find writing from the same author on different topics.

Luyckx and Daelemans [17] observed that when a large number of candidate authors were considered, similarity-based methods (ie, an anonymous document is attributed to that author whose known writing is most similar) are more appropriate than classification methods (eg, the known writings of each candidate author are used to construct a classifier which is used to classify anonymous documents). We note, however, that similarity-based methods can be best applied to text within the same medium (eg, messages from medical forums), but might not work as well for text harvested from different mediums (eg, electronic health records vs forum messages).

### Character-Based Text Classification Methods

The task of text classification consists of assigning a given text into predetermined categories. Most text classification methods are word-based (eg, they present a text document as a vector of words). In contrast, compression-based classification methods use characters or even bytes as the text representation unit. Researchers have noted that character-based classification methods have a potential advantage over word-based methods because they are able to automatically capture document features other than words. Character-based classification analyzes the text for letter counts, capitalized letters, punctuation and other nonalphabetical character counts, and letter combinations of various lengths [16,35,36]. Other important lexical features include prefixes and suffixes [18], functional words [33], and character n-grams [15]. Experiments demonstrated that letter-based methods yielded more precise results than those based on grammatical information [16].

One classification approach that has been used is compression. Having an anonymous document and several groups of documents representing several classes, a copy of the anonymous document is added to every group of documents. Each of these groups with the added anonymous document is compressed separately. As a result, the anonymous document is compressed differently with different classes of texts because the specific statistical model is created for each class of text. The document is attributed to the class that provides its maximum compression measured in bytes. The maximum compression means that the anonymous document is the most similar to the documents in this class and the created statistical model is the best for it. A relative disadvantage of this algorithm is its comparative slowness.

The most straightforward compression-based method of text categorization using off-the-shelf algorithms was described in Kukushkina et al [16]. The main idea behind this approach is that for every text the compression algorithm creates an individual model adapted to this particular class of texts. Marton et al [37] experimented with 3 compression algorithms, the data compression file format RAR, gzip, and Lempel-Ziv-Welch (LZW) [38], several corpora and types of classification, including the authorship attribution task. The attribution was performed on The Federalist Papers from the Gutenberg Project corpus [39] and a Reuters subcorpus. RAR obtained the best results compared with the other compression algorithms, with 78% overall accuracy for the Reuters corpus, which consisted of smaller texts than the other corpora.

### Prediction by Partial Matching

Teahan [40] applied compression-based methods to a multiclass categorization problem to find duplicated documents in large text collections. Comparing several compression algorithms, the author found that the best performance was obtained by the RAR software and the PPMD5 algorithm (84%-89% accuracy for different conditions). Prediction by partial matching (PPM) is an adaptive finite-context method for text compression. It is based on probabilities of the upcoming characters depending on several previous characters. These several previous characters are called “context” of the upcoming character.

Since the algorithm was first presented [41,42], it has been modified and optimized. PPM has set the performance standard for lossless compression of text throughout the past decade. It has been shown that the PPM scheme can predict English text almost as well as humans [40]. The PPM technique blends character context models of varying length to arrive at a final overall probability distribution for predicting upcoming characters in the text. The blending method is similar to the linear interpolation method of n-gram probabilities smoothing. Several methods of interpolation have been proposed [43-46].

An example of the general method of context probability interpolation is provided in Multimedia Appendix 1.

The maximal length of a context equal to 5 in the PPM model was proven to be optimal for text compression [40]. In other experiments, length of character n-grams used for text classification varied from 2 [16] to 4 [21] or a combination of several lengths [34]. Stamatatos [19] pointed out that the best length of character n-grams depends on different conditions and varies for different texts.

The PPM algorithm uses an escape mechanism for blending context probabilities. The algorithm attempts to estimate the probability of an upcoming character by using the maximal context. If this context was not found during training, then the algorithm moves to the shorter context through a so-called escape mechanism in which a probability of escape from the longer context to the shorter one is estimated and added to the final probability. If the probability of the shorter context is equal to zero, the algorithm escapes to the next shorter one and so on. If no one context is found, the algorithm estimates the probability of the upcoming character with the zero context. Given that the maximal context in our experiments is equal to 5, the full name of the method used by Authorship Attributor is PPM5. We provide the specific details of the PPM5 method in Multimedia Appendix 2.

In Bratko and Filipic [38,47], the letter-based PPM models were used for spam detection. In this task, there existed 2 classes only: spam and legitimate email (ham). The created models showed strong performance in a Text Retrieval Conference competition, indicating that data compression models are well-suited to the spam filtering problem.

In Teahan et al [48], a PPM-based text model and minimum cross-entropy as a text classifier were used for various tasks; one of them was an author detection task for The Federalist Papers. The results supported the claim made by historians and other analysts that James Madison had written the disputed papers. The modeling part of the PPM compression algorithm was used to estimate the entropy of text. The entropy provides the estimation of probabilities quality measure; the lower entropy is, the better probabilities are estimated.

In Bobicev and Sokolova [49], the PPM algorithm was applied for text categorization in 2 ways: on the basis of characters and on the basis of words. Character-based methods performed almost as well as SVM, the best method among several machine-learning methods compared in Debole and Sebastiani [50] for the Reuters-21578 Text Categorization Collection corpus.

### Comparison With Other Classification Methods

A variety of machine-learning methods have been used for text categorization, including Bayesian classification [6], decision trees [18], cluster classification [15], k-nearest neighbor (k-NN) algorithms [5], and neural nets [20]. Lately, SVM has become the most popular technique [14]. As previously described, words were the most common feature used by these methods in text classification. To put PPM classification in perspective, specifically the PPM5 model used by Authorship Attributor, we compared its performance with the performance of more standard methods.

First, we applied the word-based PPM classification [51]. Here, punctuation marks and other nonalphabetic symbols were eliminated and all letters were converted to lowercase. We used the same set of authors, texts, and other experiment settings to make direct comparison of the results: 10-fold cross-validation, 90 files for training, and 10 files for testing each time.

Next, we applied WEKA’s Naïve Bayes and SVM algorithms as the 2 most popular methods in text classification. These algorithms are able to work with various features extracted from texts. The main features in most cases were frequent words. Therefore, we used 3845 words with frequencies of more than 10 in the frequency dictionary of all words appearing in the forum texts. Because we extracted from the text words only without figures and punctuations, we added 24 features with punctuations, and also features with figures and capital letters. We then ran the classification experiment with this set of features on an in vitro fertilization (IVF) support website. The feature set “frequent words + punctuation + figures + capital letters” was built to match PPM features.

### Empirical Evaluation

#### Medical Forums

The IVF.ca website includes 8 forums: Cycle Friends, Expert Panel, Trying to Conceive, Socialize, In Our Hearts, Pregnancy, Parenting, and Administration. Table 1 presents the statistical data about the forums. Each of these forums have subforums; for example, the Cycle Friends forum consists of 6 subforums: Introductions, IVF/FET/IUI Cycle Buddies, IVF Ages 35+, Waiting Lounge, Donor & Surrogacy Buddies, and Adoption Buddies (see the summary of these in Table 1). Each of these subforums consists of a number of topics initiated by one of the participants. For example, the IVF Ages 35+ subforum consists of 506 topics such as “40+ and chances of success,” “Over 40 and pregnant or trying to be,” etc. Depending on the topic itself and the amount of interest among participants, a different number of replies are associated with each topic. For example, the former topic has 4 replies and the latter topic has 1136 replies.

For our experiments, we wanted to analyze texts authored by many forum users. Ideally, the number of texts written by each author should be as large as possible. We focused on the subforums IVF/FET/IUI Cycle Buddies [52] and IVF Ages 35+ [53] because they have the highest number of posts per author. For IVF Ages 35+ the average number of posts per author was 97.6; for the IVF/FET/IUI Cycle Buddies, the average number of posts per author was 137.8. Another important criterion for the subforum selection was the average number of posts per topic (see Table 1). Analysis showed that a topic was usually discussed through messages posted as responses to other posts on the same topic. We assumed that longer threads of topics were indicators of more posts written by the same author.

**Table 1:** Statistics on the analyzed subforums on the IVF.ca website at the time of data collection.

Subforum name	Topics, n	Posts, n	Posts per topic, mean
Introduction	1716	13,569	7.91
IVF/FET/IUI Cycle Buddies	2167	116,994	53.99
IVF Ages 35+	506	16,362	32.34
Waiting Lounge	418	3816	9.13
Donor & Surrogacy Buddies	893	7381	8.27
Adoption Buddies	304	4210	13.85

### Text Retrieval

We designed a Web crawler to retrieve messages from the Web forums and applied it to the 2 subforums mentioned previously. The 3 main stages in retrieving information using a crawler consist of (1) fetching a website, (2) parsing the HyperText Markup Language (HTML) contents of Web pages within that site, and (3) storing the retrieved data into a database. We used a combination PHP, Apache Server, and MySQL database management system in our design.

Data from each post consisted of forum name, subforum name, topic title, post author name, post author role, post date, and post content. Our code parsed the HTML contents to obtain each of these components corresponding to a given post and placed each component in a corresponding table column in the database. Post content data were used for the subsequent experiments.

### Message Preprocessing

**Figure 1:**
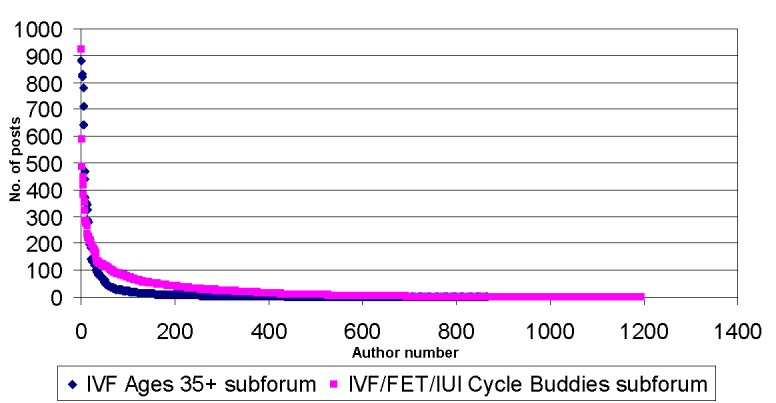
Number of posts per author distribution for the selected subforums, IVF Ages 35+ (n=865) and IVF/FET/IUI Cycle Buddies (n=1195).

We grouped posts by authors to see how much text each author produced. We sorted the data about authors by the number of posts written by each author in descending order. In total, 865 authors posted in the IVF Ages 35+ subforum and 1195 authors posted in the IVF/FET/IUI Cycle Buddies forum. The numbers of posts per author distributions for both subforums are presented in Figure 1.

**Figure 2:**
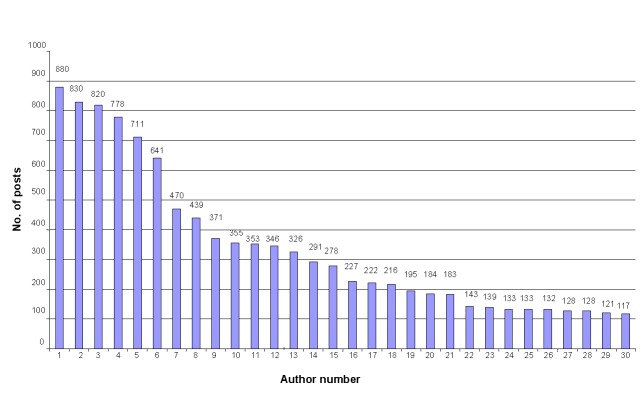
Distribution of the number of posts per author (most prolific) for IVF Ages 35+ subforum (n=30).

**Figure 3:**
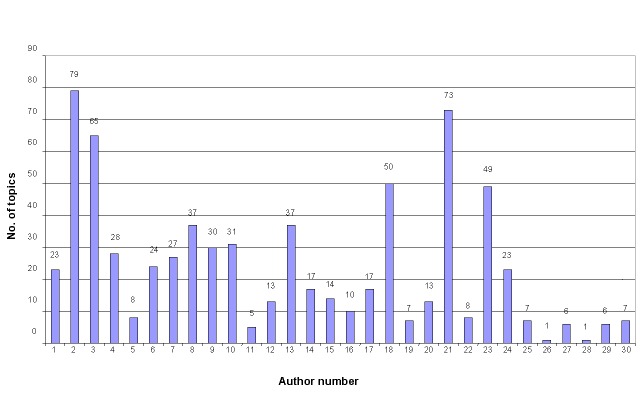
Distribution of the average post length (number of words) for the 30 most-prolific authors in the IVF Ages 35+ subforum.

We wanted to analyze as many authors as possible. At the same time, we wanted these authors to have enough posts for meaningful results. Hence, it was a trade-off between the number of authors and the number of posts, both numbers being as large as possible. For 10-fold cross-validation, 100 posts per author were enough to run machine-learning experiments [54]. In the IVF Ages 35+ subforum, 30 authors posted more than 100 messages. Statistics about the most-prolific 30 authors are presented in Figures 2 and 3, in which the total number and average length of posts for each author were measured in words (mean 126.2 words, SD 47.5). In the IVF/FET/IUI Cycle Buddies subforum, 50 authors had more than 100 posts; hence, their text volumes were larger, but the average length of posts (mean 97.7 words, SD 36) was less than that from the IVF Ages 35+ subforum.

**Figure 4:**
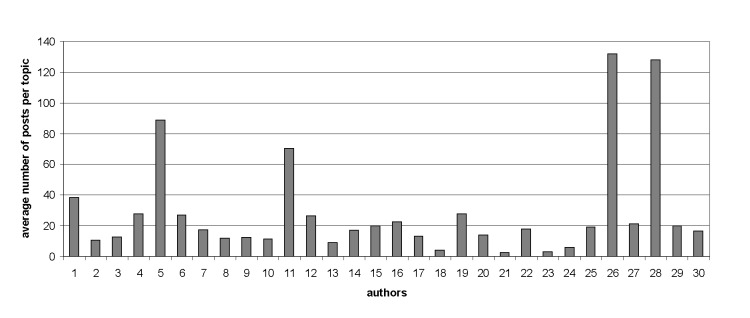
Number of posts per topic for each author (most prolific) in the IVF Ages 35+ subforum (n=30).

Figure 4 shows the number of posts per topic for each analyzed author in the IVF Ages 35+ subforum. Most authors posted approximately 10 to 20 messages on every topic. At least half of the authors posted on more than 20 topics. Such a large diversity of topics ensures that the author classification would not be influenced by the topic’s features.

The average length of posts was also important in our case because it was harder to identify the authors of shorter messages. The average length of posts was approximately 750 characters in the IVF Ages 35+ subforum and approximately 600 characters in the IVF/FET/IUI Cycle Buddies subforum. Given that the average number of characters per word on Wikipedia is 5.2 characters [55], we estimated the average length of the subforum posts to be approximately 100 to 150 words.

After examination of the data, we found that some posts included other posts; for example, “Very hot here today +40°C with the humidity. Summer is finally here! I am soooo jealous-I had my heater on in my office today!” In some cases, there were even 2 inclusions, one inside of another, so the samples of 3 author writings were mixed into 1 message. Such posts can misinform about a writing style of an individual author and should be removed from further consideration. On the IVF Ages 35+ subforum, we removed 1593 of 16,362 posts (9.74%); 14,832 posts remained for further analysis. On the IVF/FET/IUI Cycle Buddies subforum, we removed 5151 posts (15.24%); 28,640 posts remained for further analysis.

No other preprocessing of posts was necessary. Posts did not contain signatures or other personal reference to the post author. Some posts used personal names of the authors, but nicknames were used in most cases.

### Analysis

#### Experiment 1: Choice of Characters

We tested the PPM method using different sets of characters. We studied whether capitalized letters and nonalphabetic characters (eg, @,#,$,!) hold additional information about an author’s writing style.

To do this analysis, we found 60 authors who posted at least 100 messages: 30 authors from the IVF Ages 35+ subforum and 30 authors from the IVF/FET/IUI Cycle Buddies subforum. The messages from the same author represented 1 class. As a result, we had 3000 messages in the IVF Ages 35+ dataset and 3000 messages in the IVF/FET/IUI Cycle Buddies dataset. On each dataset, we ran the classification experiments by using 10-fold cross-validation. This means 10 runs of the experiment; on each run, 2700 posts were used for training and 300 posts were left for testing. Based on the cross-validation results, the confusion matrix was created and precision, recall, and *F* score were calculated [56].

Figure 2 shows that the volume of texts from the authors differed considerably. The number of posts changed from more than 800 to 100 and the average post length varied from almost 250 words for one author to less than 50 words for another. This imbalance drastically affected the results of the first experiments: the classification was biased toward classes with a larger volume of data for training. Such imbalanced class distribution problems were mentioned in previous studies [10,19,49]. Considering the fact that unbalanced data affected classification results in such a substantial way, we decided to make the data more balanced. We used 10 test texts and 90 training texts for each author, removing the additional texts from the training set.

Even in this case, we obtained an unbalanced class distribution because of different post lengths. Therefore, some normalization was necessary. We used a normalization procedure for balancing entropies of the statistical data models. The normalization procedure goes as follows: In the process of training, statistical models for each class of texts were created and probabilities of text elements were estimated. The next step after training was calculation of entropies of test documents on the basis of each class model. We obtained a matrix of entropies (class statistical models × test documents). The columns were entropies for the class statistical models and rows were entropies for test documents. After this step, the normalization procedure was applied. The procedure consisted of several steps: (1) mean entropy for each class of texts was calculated for each column of the matrix, and (2) each value in the matrix was divided by the mean entropy for this class. Thereby we obtained more balanced values and classification improved considerably. We used normalization in all the PPM5 experiments.

#### Experiment 2: Attributing Posts From Different Subforums

Machine-learning methods work better on the same types of texts; for example, Koppel et al [21] who analyzed cross-topic author identification. We ran experiments on texts posted by the same author on different subforums. In these experiments, we analyzed all authors who posted in more than 1 subforum. For each author, we extracted training texts from 1 subforum and test texts from the other subforums. We found 9 authors with at least 90 posts in 1 subforum (used for training) and at least 10 posts in other subforums (used in test) and 1 author with 88 posts in the same subforum and more than 10 posts on other subforums. These 10 authors were included in the experiment. In Table 2, we show the statistics for the authors and distribution of their posts per subforums.

**Table 2:** Statistics for authors and distribution of their posts per subforum.

Author	Subforum, n		
	Introduction	Cycle_Buddies	Age_35+
Author 1	3	6	278
Author 2	35	445	1
Author 3	7	91	3
Author 4	30	11	69
Author 5	6	88	30
Author 6	67	264	4
Author 7	13	16	820
Author 8	54	94	1
Author 9	8	7	355
Author 10	5	130	6

#### Experiment 3: Important Data Factors

##### Overview

We tested what data factors affected the accuracy of author recognition. Keeping the method and the post representation constant, we analyzed 3 data factors deemed to be important: the number of authors, the volume of training data, and the volume of test texts.

##### Number of Candidate Authors

In this set of experiments, we investigated dependence between the number of candidate authors and the accuracy of the authorship identification. We again used 100 posts for each author, splitting them 10 posts for testing and 90 posts for training in 10-fold cross-validation. For both subforums, we repeated the experiments starting with 10 authors and adding 5 authors per iteration. For the IVF Ages 35+ subforum, we had a limit of 30 authors, whereas for the IVF/FET/IUI Cycle Buddies subforum we had a limit of 50 authors.

##### Volume of Training Data

Training data volume was considered one of the most influential parameters in machine-learning methods. This experiment analyzed how accuracy depended on training data volume. We used mixed candidate authors from both subforums. We selected the first 30 authors [54] from the joint list and used 200 posts for each author. First, 20 posts were used for testing and 180 posts were used for training. Then, for each author, we reduced the number of training posts by 20, repeating the reduction until we reached only 20 training posts per author. The remainder of the settings were the same as in previous experiments: 10-fold cross-validation and PPM5 method using all characters including capitalized letters.

##### Size of Test Texts

The last critical factor was test text size. As described previously, we considered every post as an independent text author who should be detected. Some posts were really short, containing less than 5 words. Such posts were impossible to classify. Thus, we decided to unify test text sizes. We merged all test texts into 1 text and then split this text in equal fragments measuring their length in words. These experiments were performed with a mixed list of authors from both subforums created for the previous experiment. We only used the first 30 authors with the largest volume of text in their posts. All authors had at least 200 posts. In each of 10 experiments of cross-validation, we used 160 files as a training set and the remaining 40 files of the test set were merged and divided in equal fragments of specified number of words. We repeated the experiments changing test text length starting with 25 words, adding 25 more words each time until the test text reached 500 words per author.

#### Experiment 4: Comparison With Other Classification Methods

We compared PPM5 results with the results obtained by running Naïve Bayes and SVM algorithms. Both algorithms are often used in text classification and authorship attribution [15,49].

### Performance Measures

In text classification, effectiveness is measured by a combination of precision and recall. Precision is the percentage of documents classified into a category that indeed belong in that category, calculated as precision = true positive/(true positive + false positive), where true positive is the number of documents classified into a category that indeed belong to that category and false positive is the number of documents classified into the category that do not belong to that category.

Recall is the percentage of documents belonging to a category that are indeed classified into that category, calculated as recall = true positive/(true positive + false negative), where false negative is the number of documents that indeed belonged to the category but were not classified into the category [57].

The balanced *F* score is the harmonic mean of precision and recall, calculated as *F* score = 2([precision × recall]/[precision + recall]).

When effectiveness is computed for several categories, the results for individual categories can be averaged in several ways [58]: microaveraging (eg, global average of *F* score regardless of topics) or macroaveraging (eg, average of *F* scores of all topics). In our experiments, we calculated the macroaveraged *F* score.

### Generalization of Results

We estimated the significance of the PPM5 results (precision, recall, and *F* score) by computing the *t* test against those measures obtained by Naïve Bayes and SVM. Every method comparison was done on the empirical results obtained on the same forum data. Hence, we applied the paired *t* test, which is more rigorous than the unpaired version.

## Results

### Experiment 1: Choice of Characters

We first report on accuracy of the attribution from IVF Ages 35+ subforum. We used data from 30 authors, 100 posts for each author, and ran 10-fold cross-validation, 90 training and 10 test messages for each fold, to select the best performance. We investigated the impact of letter-based and character-based methods, including original capitalization and conversion to lower case. The results reported in Table 3 show character-based PPM performed better when it worked with all the characters including capitalized letters.

The same experiments were conducted on the base of IVF/FET/IUI Cycle Buddies subforum posts using 100 posts for each of 30 selected authors. The results are presented in Table 4.

### Experiment 2: Attributing Posts From Different Subforums

To obtain results on the IVF Ages 35+ subforum using the word-based PPM classification model, we used 1 run of the classifier training and then tested the classifier on the test set. We used 90 training posts from 1 subforum and 10 test texts collected from other subforums. The results are: precision = 0.822, recall = 0.810, *F* score = 0.816. A slight decrease in *F* scores can be explained by the small number of posts. In many cases, the posts were extremely short, especially the test ones, and this affected the results.

### Experiment 3: Important Data Factors

#### Effect of Number of Authors

**Figure 5:**
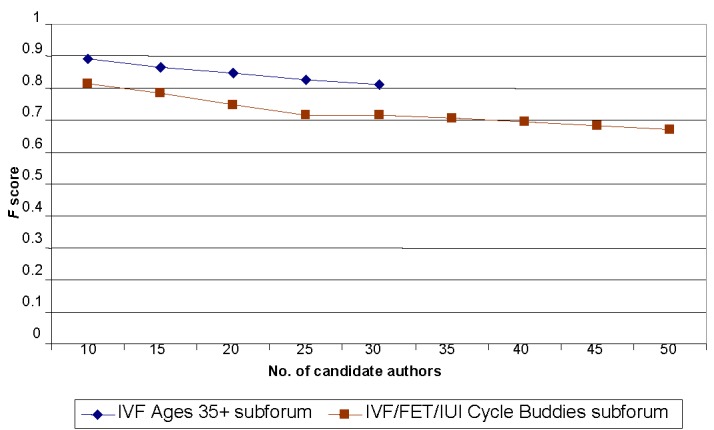
Dependency of the accuracy on candidate author number for author detection task on the IVF/FET/IUI Cycle Buddies and IVF Ages 35+ subforums.

We used the same dataset as for the rest of our experiments: 100 posts for each author, 10 for testing, 90 for training, 10-fold cross-validation. For both subforums, we repeated the experiments changing the number of authors. Tables 5 and 6 present the results for both subforums. Figure 5 demonstrates the dependencies between the number of authors and the accuracy of the attribution.

**Table 3:** The IVF 35 Ages + classification results; 10-fold cross-validation, 30 authors, 100 posts per author.

Model	*F* score	Precision	Recall
Letters	0.793	0.803	0.784
Characters lowercase	0.822	0.830	0.831
Original capitalization	0.826	0.836	0.817

**Table 4:** Classification results for author identification on IVF/FET/IUI Cycle Buddies subforum; 10-fold cross-validation, 30 authors, 100 posts per author.

Features	*F* score	Precision	Recall
Letters	0.836	0.851	0.822
Characters lowercase	0.887	0.896	0.877
Original capitalization	0.902	0.911	0.894

**Table 5:** Dependency of the accuracy of author detection task on candidate author number on the IVF/FET/IUI Cycle Buddies subforum.

Number of authors	*F* score	Precision	Recall
10	0.965	0.967	0.963
15	0.932	0.937	0.927
20	0.924	0.931	0.917
25	0.912	0.921	0.904
30	0.902	0.911	0.894
35	0.881	0.891	0.872
40	0.845	0.856	0.835
45	0.838	0.849	0.827
50	0.831	0.842	0.820

**Table 6:** Dependency of the accuracy of author detection task on candidate author number on the IVF Ages 35+ subforum.

Number of authors	*F* score	Precision	Recall
10	0.919	0.921	0.916
15	0.918	0.922	0.914
20	0.885	0.889	0.882
25	0.875	0.882	0.869
30	0.826	0.836	0.817

#### Effect of Size of Training Data

**Figure 6:**
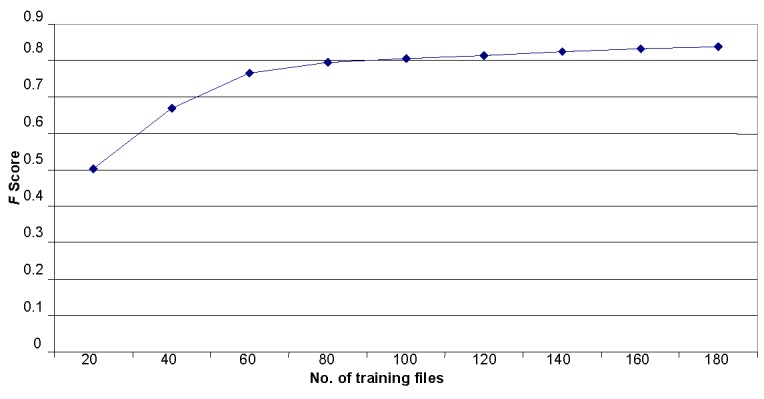
Dependency of the *F* score and the training data volume for the author attribution.

We analyzed how the attribution accuracy depended on the training data volume. The results of these experiments are presented in Table 7. The *F* score rapidly rose from 0.5 to 0.8 when the number of training texts reached 100 posts. After that, the increase in the training set did not change the *F* score. The graph in Error: Reference source not found Figure 6 visualizes the relationship between the number of training files and the *F* score.

#### Effect of Test Text Size

**Figure 7:**
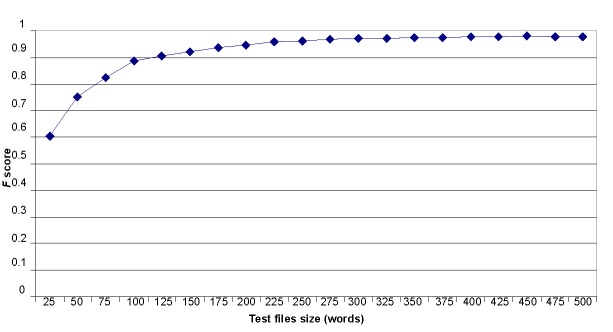
Dependency of the *F* score on the test text size for the author attribution.

We checked the impact of the test size (words) on the author attribution. Table 8 summarizes the results of the experiments. The *F* score rapidly increased with the increase of the text from 25 to 100 words, and then slowly increased until the test text reached 275 words. After that, the *F* score fluctuated, although the overall tendency was still to increase. The relationship between text size and the *F* score is shown in Figure 7.

**Table 7:** Dependency of the accuracy on training data volume for the author detection task.

Number of training files	*F* score	Precision	Recall
20	0.503	0.496	0.511
40	0.668	0.669	0.667
60	0.765	0.773	0.758
80	0.794	0.800	0.787
100	0.806	0.812	0.800
120	0.815	0.823	0.808
140	0.826	0.834	0.819
160	0.834	0.841	0.827
180	0.837	0.843	0.831

**Table 8:** Dependency of the results of test files size for author detection task.

Test files size (words)	*F* score	Precision	Recall
25	0.605	0.613	0.599
50	0.752	0.759	0.745
75	0.825	0.833	0.817
100	0.886	0.895	0.877
125	0.907	0.914	0.901
150	0.920	0.926	0.915
175	0.936	0.940	0.933
200	0.948	0.952	0.943
225	0.958	0.963	0.953
250	0.962	0.967	0.957
275	0.970	0.973	0.967
300	0.973	0.976	0.971
325	0.972	0.975	0.969
350	0.976	0.979	0.973
375	0.975	0.978	0.973
400	0.979	0.981	0,976
425	0.977	0.980	0.975
450	0.980	0.982	0.978
475	0.978	0.980	0.975
500	0.979	0.982	0.977

### Experiment 4: Comparison With Other Classification Methods

When we compared the performance of our method to other classification methods, the results were nonuniform. For the IVF Ages 35+ subforum, SVM on the most complex set of features gave the best result (*F* score=0.766). The Naïve Bayes algorithm performed better on frequent words only, but its *F* score was only 0.636. For the IVF/FET/IUI Cycle Buddies subforum, SVM again was better, but this time on frequent 5-character sequences (*F* score=0.701). The best Naïve Bayes was on frequent words only (*F* score=0.575). The *F* score obtained on different sets of features on both subforums for these methods are presented in Table 9.

The obtained results show that for authorship attribution, word-based classification is not as good as character-based classification. Also, PPM outperformed Naïve Bayes and SVM on the reported experiments for this task.

### Statistical Significance of the PPM5 Results

The *t* test results for the IVF35+ subforum show that PPM5 outperformed Naïve Bayes with a significant difference (*P*=.02, standard error of the difference=0.025). PPM5 significantly outperformed SVM (*P*=.001, standard error of the difference=0.002). The *t* test results on the IVF/FET/IUI Cycle Buddies subforum show that PPM5 significantly outperformed Naïve Bayes (*P*=.008, standard error of the difference=0.027). PPM5 significantly outperformed SVM (*P*<.001, standard error of the difference=0.001).

**Table 9:** Results for author detection task using Naïve Bayes and support vector machine (SVM) classification models implemented in WEKA.

Subforum	Features	*F* score	
		Naïve Bayes	SVM
IVF Ages 35+	Frequent words only	0.636	0.760
IVF Ages 35+	Frequent words + punctuation + figures + capital letters frequency	0.624	0.766
IVF Ages 35+	Frequent 5-character sequences	0.586	0.743
IVF/FET/IUI Cycle Buddies	Frequent words only	0.575	0.690
IVF/FET/IUI Cycle Buddies	Frequent words + punctuation + figures + capital letters frequency	0.567	0.694
IVF/FET/IUI Cycle Buddies	Frequent 5-character sequences	0.550	0.701

## Discussion

### Principal Findings

In this study, we aimed to empirically examine the accuracy of identifying authors of online posts on a medical forum. Given that individuals may be reluctant to share personal health information on online forums, they may choose to post anonymously. The ability to determine the identity of anonymous posts by analyzing the specific features of the text raises questions about health consumers using anonymous posts as a method to control what is known publicly about them. We measured the accuracy of the direct author matching for a single post that produced an *F* score of 75% to 80% on messages containing 100 to 150 words on average. On messages containing at least 300 words, we obtained an *F* score of 0.979.

The focus of this work was to show that character-based PPM5 can identify authors with a high accuracy. Given the results, we can conclude that our hypothesis was correct. We have shown that the application of PPM5 makes an automated identification of the author of an online post possible. Our method was able to correctly attribute authors with high confidence (ie, *F* score up to 0.979). PPM was demonstrated to create the best statistical text model and to predict it almost as well as humans [40].

It should be noted that the data was very unbalanced. Some authors had hundreds of posts and some had written only tens. In addition, some authors posted long texts with descriptions and discussions and some tended to post just short replies to other posts, for example, “GF - I am so sorry,” “Congrats Lisa!,” and “Saffy - I love you.” As a result, we had to apply the text normalization. The feature set is one of the most important factors in author attribution methods. PPM is character-based because it uses character n-grams as features. Although PPM could be applied on the word-based level, it was demonstrated that it did not perform better than character-based PPM for text classification tasks [51]. A number of researchers used characters and character n-grams for author detection tasks [16,35,36]. Character n-grams captured most of the features used by other methods such as prefixes and suffixes, prepositions, pronouns, conjunctions, abbreviations and other frequent words, errors and idiosyncrasies, punctuations, special symbols (eg, smiles), and others in a natural way without complex preprocessing.

In our experiments, we found evidence that all characters from the text are important for author writing style detection. The results of the experiments demonstrated that the use of different nonalphabetical characters improved the results of character-based PPM experiments.

At the beginning of our experiments, we saw that shorter messages posted on the IVF/FET/IUI Cycle Buddies subforum tended to have poorer classification results. We selected the top 100 long posts for each of the 30 analyzed authors for our experiments. Consequently, the attribution for the IVF/FET/IUI Cycle Buddies subforum improved considerably and was even better than for the IVF Ages 35+ subforum.

Concerns about topic-specific features which helped in classification but did not actually present an author’s specific writing style were expressed in some works dedicated to the authorship attribution problem [10,13,15,18]. To verify the ability of our classification method to work on different topics, we found 10 authors who posted in several subforums. We performed an experiment using training files from 1 subforum and test posts from other ones. The attribution *F* score decreased (from 0.826 to 0.816 for IVF Ages 35+ subforum). This can be explained by short posts that we had to use (eg, “Welcome, glad you found the site!”). In the previous experiments, we were able to delete such short posts; in this one, we did not have enough posts to do this.

Comparisons with the other classification methods demonstrated that the character-based PPM method gives the best results: the *F* score for IVF Ages 35+ subforum was equal to 0.826 with use of nonalphabetical symbols and capitalized letters. The application of word-based PPM, Naïve Bayes, and SVM on the same subforum did not show as good results as the character-based PPM; for example, the best *F* score of 0.766 was obtained by SVM. To evaluate the overall performance of the algorithms, we analyzed the significance of the difference between the PPM5 results and those of Naïve Bayes and the PPM5 results and those of SVM. We applied paired *t* tests and showed that on the data gathered from each subforum and for the all algorithm pairs, the difference is statistically significant.

There were 3 strongly influencing factors in author classification: (1) number of candidate authors, (2) volume of training data, and (3) the size of test text. We analyzed the 3 factors using the data from the 2 subforums.

First, we increased the number of authors from 10 to 30 for the IVF Ages 35+ and from 10 to 50 for the IVF/FET/IUI Cycle Buddies. The main conclusion was that the method was able to handle more authors with a comparatively little loss in accuracy; the author was detected correctly for more than 90% of posts with 10 candidate authors, and we had less than 10% loss of accuracy for 30 authors. Further increase in the number of authors to 50 again decreased the accuracy by less than 10%. The decrease depended on the authors added or removed from the experimental set. Some authors tended to write comparatively long messages and their posts were easier for the method. There were some authors who tended to write a lot of short replies to other posts (10-20 words) for which the accuracy of recognition was considerably lower. Even with 50 candidate authors, the *F* score was approximately 0.83. To compare with previous results for the authorship attribution, in Kukushkina et al [16], 73% accuracy was obtained on 82 literary works in a Russian authors’ corpus, but they worked with much larger volumes of training and testing texts. Luyckx and Daelemans [17] studied dependency of accuracy on number of authors and obtained 82% accuracy on 10 authors, but it had fallen to less than 50% for 50 authors.

Next, as the training data volume was considered the most influential factor in all statistical methods, we tested the relationship between accuracy and the number of files used for training, changing the latter starting with 20 files and adding each time 20 more until we reached 180 files. The *F* score grew fast for the first 100 files—from 0.50 to 0.80—and then the growth slowed down. We hypothesize that to reach *F* score= 0.90, we have to have training data 10 times more than the test data. In practice, this is hard to obtain.

In the experiments with training data volume, our best *F* score was 0.837. It was greater than in the first set of experiments. We can explain this increase by the fact that we mixed texts of 2 subforums. The content of the subforums was different and it helped to categorize messages more precisely. This approach may be helpful when we want to identify authors posting messages on various subforums.

The last factor we tested in the experiments was the test text size. Initially we considered each post as a separate test text and made all our experiments on the basis of these settings. We noticed that some posts were extremely short (3-5 words), as in examples presented previously. Thus, we made experiments with longer fragments of test texts. Even 25-word messages were recognized with an *F* score higher than 0.60 and it grew until message length reached 300 words. The *F* score actually remained the same (approximately 0.97) for messages with lengths from 300 to 500 words. We can conclude that this was the accuracy limit for this method and it was reached for messages with the length at least 300 words.

Based on the reported study and obtained empirical evidence, we have concluded that authorship can be successfully detected in free-form messages posted on medical forums.

### Limitations

We focused exclusively on IVF forums in this study; therefore, our results are limited to the IVF context. It is unclear whether these results can be generalized to forums focusing on different topics (eg, smoking cessation, heart disease, cancer). Research on different forum topics should be conducted to expand these results further.

Also, it is unclear whether the results from IVF forums would be useful in identifying anonymous users posting on other forums (eg, smoking cessation forums). Certain text features may be specific to a topic and may not be useful in identifying anonymous authors across forums of varying topics.

### Practical Implications

The main implication of our results is that they should caution users from posting sensitive information anonymously. Managers of online properties that encourage user input should also alert their users about the strength of anonymity. Our experiments show that a character-based method can be more effective than word-based methods in authorship attribution. These are novel results for forum analysis because the usual methods of text analysis are based on semantics and analyze the use of words, phrases, and other text segments. We propose that to improve security of forum members, the forum organizers pay more attention to the character-based characteristics of the posts.

Does this mean that posting anonymously is futile and that all consumers should just use their real identity? Moving forward, this is not necessarily the case. Future work can extend tools such as Authorship Attributor to (1) alert anonymous posters about the ease of determining their identity so they can then make a more informed decision about the content of their posts (eg, by informing consumers with many posts on the same topic that they will have a higher chance of being reidentified through their posts than those with fewer posts on many diverse topics), and (2) automatically modify the text to adjust its features to make it correlate less with other text from the same author and, hence, frustrating tools such as Authorship Attributor.

## Multimedia Appendix 1

An example of the general method of context probability interpolation.

## Multimedia Appendix 2

Details of PPM5 method.

## Abbreviations

HTML: HyperText Markup Language
IVF: in vitro fertilization
PPM: prediction by partial matching
SVM: support vector machine
