# Supplementary material for: Can Anonymous Posters on Medical Forums be Reidentified?
Source: J Med Internet Res. 2013 Oct 3;15(10):e215. doi: 10.2196/jmir.2514 (PMC3806358; doi:10.2196/jmir.2514)
Supplement: Supplementary file 1 [file jmir_v15i10e215_app1.pdf]

Multimedia Appendix 1

Context Probability Interpolation

An example of the general method of context probability interpolation is the probability of character “l” in the context of the word medical calculated as a sum of conditional probabilities of this character in dependence of different context length up to the limited maximal length, in this particular case equal to 5:

P_{blended} (‘l’) = $\lambda_5 \cdot P$(“l” | “edica”) + $\lambda_4 \cdot P$(“l” | “dica”) + $\lambda_3 \cdot P$(“l” | “ica”) + $\lambda_2 \cdot P$(“l” | “ca”) + $\lambda_1 \cdot P$(“l” | “a”) + $\lambda_0 \cdot P$(“l”)

where λ_i (i = 1...5) are normalization coefficients; some of them can be equal to zero and $\sum_{i=1}^{5} \lambda_i = 1$, where 5 is the maximal length of the context.
