# Supplementary material for: Can Anonymous Posters on Medical Forums be Reidentified?
Source: J Med Internet Res. 2013 Oct 3;15(10):e215. doi: 10.2196/jmir.2514 (PMC3806358; doi:10.2196/jmir.2514)
Supplement: Supplementary file 2 [file jmir_v15i10e215_app2.pdf]

Multimedia Appendix 2

As a compression algorithm, PPM is based on the notion of entropy introduced as a measure of a message uncertainty [1]:

$$H_d = -\sum_{i=1}^{n} p(x_i) \log p(x_i)$$

where H_d is the entropy of text *d*; $P(x_i)$ is the probability of character x_i (i = 1...n) for all characters in the text *d*.

In practical tasks, the per-character entropy is used:

$$H_L = \frac{1}{n}\left(-\sum_{i=1}^{n} p(x_i) \log p(x_i)\right)$$

where probabilities of the characters can be estimated from the same text or from other texts.

Cross-entropy is the entropy calculated for a text if the probabilities of its characters have been estimated on another text [2]:

$$H_d^m = -\sum_{i=1}^{n} p^m(x_i) \log p^m(x_i)$$

where $H^m{}_d$ is text *d*'s entropy obtained by the model *m*; $p^m(x_i)$ - probability of character x_i using model *m* for all characters in the text *d* (*i* = 1...*n*) *m* is a statistical model created on the base of another text. The cross-entropy between 2 texts is greater than the entropy of a text itself, because probabilities of characters in diverse texts are different:

$$H_d^m \geq H_d$$

Cross-entropy can be used as a measure of document similarity: the lower the cross-entropy for 2 texts is, the more similar they are. Cross-entropy can be used for text classification when several statistical models had been created using documents that belong to different classes and cross-entropies are calculated for an unknown text based on each model, the lowest value of cross-entropy will indicate the class of the unknown text.

References

1. Katz S. Estimation of probabilities from sparse data for the language model component of a speech recognizer. IEEE Transactions on Acoustics, Speech and Signal Processing 1987 Mar. 35(3):400 – 401. DOI: 10.1109/tassp.1987.1165125
2. Teahan W. Modelling English text. PhD Thesis, University of Waikato, New Zealand, 1998.
